# The Structure Beneath the Symbols: How Children Develop an Understanding of Place Value

**DOI:** 10.1177/09637214261417968

**Published:** 2026-03-17

**Authors:** Chuyan Qu, Daniel Ansari

**Affiliations:** 1 Centre for Brain and Mind, Western University; 2 Faculty of Education, Western University

**Keywords:** place-value understanding, numerical cognition, developmental psychology, learning, compositional structure

## Abstract

The place-value concept is fundamental to understanding the symbolic number system. It dictates that the value of a digit in a number is based on its position or place within the number (e.g., the “5” in “510” is five units of 100, whereas the “5” in “51” is five units of 10). Place value is central to understanding multidigit numbers, performing arithmetic, and learning more complex math. Despite its significance, relatively little research has systematically examined the developmental trajectory and cognitive underpinnings of the place-value concept. In this article, we synthesize prior findings and propose a conceptual framework that delineates the core properties of the place-value concept and characterizes its developmental trajectory. We also identify key cognitive factors that may underpin individual differences in its acquisition. This framework can guide future research to understand how children acquire the place-value concept and how best to support this learning. It also has broad implications for understanding the cognitive architecture of human compositional symbol systems.

The invention of compositional symbol systems stands as one of the most remarkable achievements of human culture. In such systems—natural language being a prime example—a finite set of symbols is systematically combined according to a finite set of syntactic rules, enabling infinite expressive capacity. The Arabic numeral system is another example of such a compositional structure: It comprises only 10 distinct symbols (0–9) yet encodes an infinite range of numerical values through the application of the place-value concept. The place-value concept dictates that the value of a digit in a numeral is based on its place within the numeral. Having an explicit understanding of place value means knowing that, for example, the “5” in “564” is five units of 100, that the “6” is six units of 10, and that the “4” is four units of one. The place-value concept serves as the basis for the comprehension and production of large numbers, multidigit arithmetic, and more advanced mathematical skills (Herzog & Fritz, 2022; Ho & Cheng, 1997; McCloskey, 1992; Moeller et al., 2011).

Young children often struggle with understanding place value—a difficulty that can persist into the upper elementary grades and is linked to math difficulties and poor long-term outcomes (Chan & Ho, 2010; Fuson, 1990; Fuson & Briars, 1990; Hanich et al., 2001; Jordan & Hanich, 2000; Lambert & Moeller, 2019; Ross, 1986). Therefore, understanding the developmental processes and cognitive underpinnings of children’s acquisition of the place-value concept is crucial.

The significance of place value extends beyond learning multidigit numbers. Place value is one of the earliest and most consequential culturally invented symbolic systems shaped by a long and complex historical evolution (Zhang & Norman, 1995). Unlike innate numerical abilities, place value combines arbitrary written symbols, spatial conventions, and compositional rules to express an unbounded set of numerical meanings. As such, children’s early difficulties with place value are not merely errors in numerical reasoning but reveal deeper questions about how learners come to understand complex representational systems and how cultural conventions become cognitively internalized. Thus, place value offers a powerful window into the cognitive foundations of human compositional symbol systems more broadly.

Here, we describe what is known about early place-value understanding^1^ and how it has been investigated by cognitive scientists and educators. We first delineate the essential properties of the place-value system. On this basis, we then introduce a conceptual framework to characterize its developmental trajectory and identify key cognitive mechanisms that may underlie individual differences in its acquisition.

## What Are the Properties of Place Value?

The place value system has three key properties that are crucial for understanding multidigit numbers:

1. *Positional principle*. The value of an individual digit is determined by its position within the number, eliminating the need for labels such as “hundred” or “thousand” (Cheung & Ansari, 2021; Ross, 1989). The same digit can have different values depending on its position—for example, in the number “333,” the digits represent 300, 30, and 3, respectively. Under this principle, zero plays a critical role as a placeholder. Unlike spoken number words in which the zero is often omitted (e.g., “three hundred and two”), the written numeral “302” includes a zero to preserve the correct positional values. The positional principle highlights the relation between position (or place) and value and forms the foundation of place-value understanding (Cheung & Ansari, 2021).
2. *Base-ten rules*. In the base-ten system, each digit place can be filled by one of 10 digits (0–9), and each place represents increasing powers of 10 from right to left: 10^0^, 10^1^, 10^2^, 10^3^, and so on (e.g., the hundredth place represents 10^2^).
3. *Compositional structure*. The compositional structure of the place value consists of both additive and multiplicative properties (Ross, 1989). The value of an individual digit is determined by multiplying its face value by the corresponding base (multiplicative property), whereas the overall value of the numeral is the sum of the values of its digits (additive property). For example, “834” represents (8 × 100) + (3 × 10) + (4 × 1).

## How Does Place-Value Understanding Develop?

The development of place-value understanding is a complex process unfolding from emerging partial knowledge to a more sophisticated grasp of the base-ten structure (Bower et al., 2022; Byrge et al., 2014; Mix et al., 2014, 2022). Yet the fine-grained developmental changes that occur as children reach school age remain understudied. In this section, we draw on prior research to propose a conceptual framework that characterizes the developmental trajectory of place-value understanding (see also Cheung & Ansari, 2021; Fuson et al., 1997; Herzog et al., 2019; Ross, 1986; Sinclair et al., 1992). This framework proposes that six components constitute the development of the place-value concept. We discuss these components in turn in the sections that follow.

### Multidigit numbers as strings

Multidigit numerals are common in children’s environments (e.g., room numbers, phone numbers, street addresses). As hypothesized by Mix et al. (2014), children may notice statistical patterns in these number strings that can help them infer the numerical meanings of multidigit numbers. We propose that children may initially treat multidigit numbers as mere strings of digits. Even 3-year-olds can compare the numerical magnitude of multidigit numbers in written form (Mix et al., 2014), but their above-chance performance could be consistently explained by heuristic strategies based on the perception of digit strings. For example, children may infer that visually longer digit strings represent larger values (e.g., judging 100 as greater than 10). When comparing numbers with the same length, children prefer those containing larger individual digits—such as choosing 223 over 220.

### Multidigit numbers as whole units

At around 4 years of age, children begin to view multidigit numbers as unified wholes (cardinalities) rather than collections of separate digits (Fuson & Briars, 1990; Ross, 1986; Sinclair et al., 1992). Yuan et al. (2019) suggested that some children as young as 3 may already recognize that a number such as 65 is not simply a six and a five that are close together. Yet, at this stage, the parts of the multidigit have no clear or discernible meanings. Children do not grasp that a digit’s position indicates its value (Sinclair et al., 1992). For instance, they may interpret “23” as the spoken label “twenty-three,” which represents some amount without recognizing that the “2” represents two tens and the “3” represents three ones. This may help explain findings by Mix et al. (2014) that revealed that children’s ability to map spoken number names onto written multidigit numbers improves more rapidly and steadily between the ages of 3 and 4 than their ability to determine which digit represents a larger value.

### Developing knowledge of the positional principle

A key developmental milestone in place-value understanding is grasping the principle of position, recognizing that digits in different positions represent ordered values of different magnitudes (Cheung & Ansari, 2021). Cheung and Ansari (2021) demonstrated that children begin forming this connection between place and value at around age 6. This emerging understanding is often reflected in their ability to compare numbers with reversed digits (e.g., recognizing that 62 is greater than 26) even before they fully grasp the rules of the base-ten system. For instance, a child may understand that the digit “6” in “62” holds a larger value than in “26” because it appears in the leftmost position. However, they often cannot specify what that position represents (e.g., that the “6” in “62” represents six tens). Furthermore, when children younger than 6 are asked to map a two-digit numeral onto the quantity it represents, they typically perform at or below chance level, suggesting they lack an intuitive sense of the exact physical quantities represented by multidigit numbers (Yuan et al., 2019).

### Developing knowledge of independent place-value units

When children are formally introduced to place-value terminology in elementary school (typically around ages 6–9), often through base-ten blocks, they begin to learn that each digit in a multidigit numeral represents an independent unit tied to its position (Fuson et al., 1997; Ross, 1986). In the case of a two-digit number, they may attach the label “tens” to the left digit and “ones” to the right digit. Their understanding of place value is often superficial, lacking awareness of its compositional structure (see above)—that ten ones can be regrouped into one ten, or that one ten can be decomposed into ten ones. These limitations are especially apparent in tasks requiring regrouping. For example, when asked to interpret the digits in “42” using a noncanonical representation (e.g., three ten-blocks and 12 unit-blocks), children must mentally regroup the blocks to recognize that the “4” represents four tens—including one formed from the 12 units—and the “2” represents the remaining two ones. At this stage, many children struggle to apply the tens-for-one trading principle (Fuson, 1990; Ross, 1986). A limited understanding of the compositional structure is associated with persistent difficulties in arithmetic, especially with carrying and borrowing (Fuson, 1990; Mix et al., 2023; Moeller et al., 2011).

### Developing knowledge of integrated place-value units

With formal instruction in elementary school, children begin to develop a more integrated understanding of the place-value system (e.g., Fuson et al., 1997; Herzog et al., 2019). They understand that 10 units in one place can be exchanged for one unit in the next higher place (e.g., ten ones can be exchanged for one ten), and vice versa. This understanding extends beyond two-digit numerals and generalizes to numbers with three or more digits (Herzog et al., 2019). At this stage, children’s success in noncanonical regrouping tasks reflects their ability to actively apply the compositional base-ten structure by composing and decomposing quantities across place-value units (Moeller et al., 2011; Ross, 1986). This ability is essential for syntactic understanding of place value and for achieving fluency in multidigit arithmetic (Fuson et al., 1997).

### Advanced understanding of the compositional syntax of place value

With more advanced mathematical instruction, such as the introduction of exponential notation, we hypothesize that students can easily translate their understanding of the base-ten system into formal expressions. For example, they can represent the number “834” as (8 × 10^2^) + (3 × 10^1^) + (4 × 10^0^). At this level, students can also generalize this compositional structure to other base-*N* systems. For instance, they can flexibly convert numerals from a base-two (binary) system into their base-ten equivalents.

## Cognitive Underpinnings of Place-Value Understanding

**Fig. 1.**
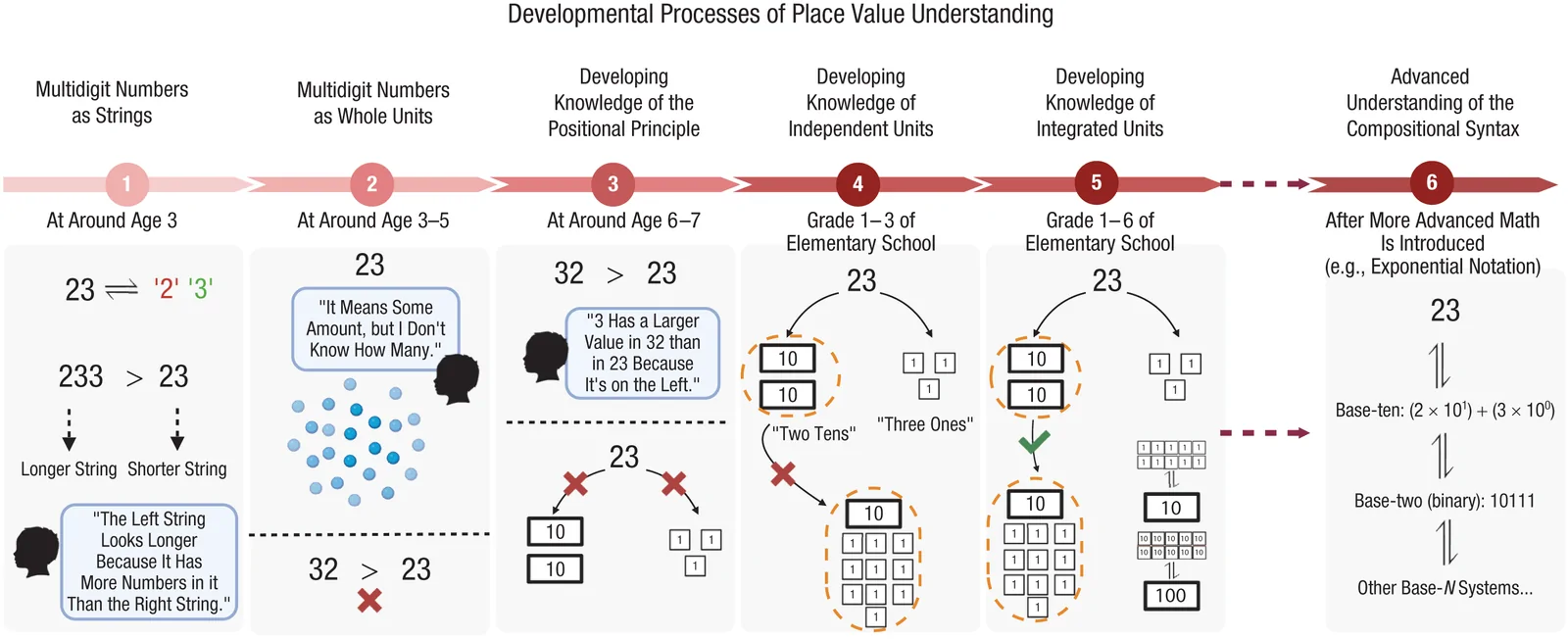
Proposed framework for the developmental processes of place-value understanding. The proposed trajectory is based on currently available evidence but should not be interpreted as the definitive developmental pathway for all learners. Age ranges indicate when particular forms of place-value knowledge typically begin to emerge. The red Xs denote competencies that have not yet developed at a given profile.

Elucidating the cognitive underpinnings that drive developmental changes in place-value knowledge and that account for individual differences in later mastery is critical for a mechanistic account of place-value learning. Here, we use “cognitive underpinnings” to refer to domain-specific cognitive components that support the development of place-value concepts (e.g., early knowledge of multidigit numerals). Although these domain-specific components may interact with broader domain-general skills (such as attention or working memory), our focus in this section is on the domain-specific knowledge components that serve as foundations for place-value understanding, enabling children to progress along the developmental pathway (Fig. 1).

First, on the basis of previous research, children’s early but imperfect knowledge of multidigit numerals (i.e., how to write, read, and count numbers) provides an entry point into understanding place value (e.g., Bower et al., 2022; Mix et al., 2022). This emerging knowledge is commonly assessed by transcoding tasks that require children to bidirectionally map between spoken and written number forms (e.g., reading the numeral “71” or writing down the heard number “twenty-three”). A longitudinal study using network analysis found that early skills in reading and writing numbers serve as a central skill that integrates pieces of knowledge about place value with base-ten principles (Bower et al., 2022). Cheung and Ansari (2021) also found that knowing number names of multidigit aids in learning the positional principle of place value, possibly because frequent exposure to number words helps children discover patterns such as the leftmost digit representing the largest value (Cheung & Ansari, 2021; Yuan et al., 2019). Additionally, “smart errors” such as writing “600405” for “645” may reflect partial knowledge of the base-ten structure and may serve as important stepping stones toward full place-value understanding (Bower et al., 2024; Byrge et al., 2014).

In addition to number-specific knowledge, several nonnumerical cognitive abilities may play a fundamental role in place-value understanding. One crucial factor we propose is children’s *ordering ability*. Spatial ordering of numbers has been associated with children’s understanding of the magnitude represented by symbolic numerals (Sella et al., 2019, 2020). Furthermore, children’s general ordering abilities, as measured by the ordering of sequences of everyday events, were significantly related to formal math skills both concurrently and longitudinally (O’Connor et al., 2018). To understand place value, children must understand that the magnitudes of the values each position represents increase in ascending order from right to left, a core feature of the positional principle.

Another crucial factor we propose is the understanding of *part-whole compositionality*. Early studies found that success on place-value tasks is associated with children’s ability to reason about part-whole relations, as measured through arithmetic word problems and logical classification tasks (Ross, 1986). This implies that understanding part-whole relations is foundational for grasping how values of individual digits contribute to the total value of a multidigit numeral and how whole numbers can be decomposed into subsets (e.g., “22” as two tens and two units). This knowledge sits at the core of the compositional structure of place value, encompassing its additive and multiplicative properties (e.g., the number “302” is equivalent to 3 × 100 + 0 × 10 + 2 × 1).

A third factor we propose that may influence place-value understanding is children’s understanding of *recursive structure*. Recursion—the repeated embedding of structures within structures of the same kind—has long been considered a foundation of human syntax and symbolic competence (Hauser et al., 2002; Pinker & Jackendoff, 2005). Evidence suggests that the ability to process recursive patterns is widespread across cultures and even present in some nonhuman species (Ferrigno et al., 2020; Liao et al., 2022). Dehaene et al. (2025) further proposed that recursive embedding may have motivated the development and cultural invention of efficient numerical notation systems. The base-ten place-value system is itself inherently recursive: It allows increasingly large numbers to be generated by repeatedly applying the similar nested structures (e.g., 10 units make one 10; 10 tens make one 100) and by reusing the same set of lexical primitives (the digits 0–9). We therefore hypothesize that early sensitivity to recursive structure may facilitate children’s acquisition of base-ten rules, enabling the “infinite generativity” of numerals from a finite set of digits.

## Conclusion and Future Directions

This article clarified key properties of the place-value concept and offered a conceptual framework to more precisely characterize the developmental trajectory of place-value understanding. We also highlighted the core cognitive mechanisms that may underlie individual differences in the acquisition of this foundational concept.

Despite this progress, several gaps remain in the literature. One major challenge concerns how place-value understanding is measured. Existing tools have been invaluable in documenting children’s progression from partial knowledge to mastery (e.g., Chan et al., 2014; Mix et al., 2022). However, we speculate that those tools often tap multiple cognitive components at once, making it challenging to determine which underlying skills drive performance at different stages. If place-value understanding is indeed multicomponent, future work will benefit from an expanded, more differentiated measurement tool kit—one that can selectively target specific subcomponents (e.g., positional principles, recursive structure) and more clearly distinguish among the developmental profiles proposed in Figure 1. Such tools would complement existing measures by enabling more rigorous tests of the structure and progression of children’s emerging place-value knowledge.

Empirical studies are also needed to investigate the cognitive components of place-value development. Longitudinal studies that measure the same variables in the same children at multiple time points, for example, could illuminate relations between key cognitive factors and the acquisition of place-value understanding. Decomposing the cognitive components of place value may inform the design of targeted educational interventions to support children’s emerging understanding of multidigit numerals.

Another promising direction is to examine the influence of educational context and linguistic environment on children’s conceptual development. Cross-cultural evidence has highlighted significant variability in how and when children grasp place value (Fuson, 1990; Miura et al., 1993). For example, regular number-naming systems in languages such as Chinese and Korean (e.g., “two-ten-one” for 21) support earlier and more robust understanding of the base-ten system. In contrast, languages such as English contain irregularities (e.g., “eleven,” “twelve”) that obscure the base-ten structure and may hinder early understanding (Miura et al., 1993; Miura & Okamoto, 1989). Beyond language, differences in curricula, instructional emphasis, and the use of manipulatives may further shape how and when children acquire place-value knowledge (Lafay et al., 2023; McGuire & Kinzie, 2013; Mix et al., 2024).

In summary, continued research in these directions promises to advance our understanding of how children acquire place-value knowledge and how best to support this learning within diverse educational contexts. The processes involved in learning place value—translating across different forms of representations (e.g., verbal, written, and quantitative) and internalizing compositional and recursive structure beneath symbols—reflect mechanisms central to symbolic reasoning. Similar processes may support learning in other domains such as algebra, geometry, musical notation, and programming languages. As a core principle of symbolic number systems, place value thus provides a powerful lens for uncovering the origins and cognitive foundations of culturally invented compositional symbol systems more broadly.

## Recommended Reading

Bower, C. A., Mix, K. S., Yuan, L., & Smith, L. B. (2022). (See References). Uses network analysis to show how formal instruction transforms children’s piecemeal multidigit knowledge into a more integrated, principle-based system.

Cheung, P., & Ansari, D. (2021). (See References). Shows that children begin to grasp the positional principle of place value around age 6 and that knowledge of number names supports this understanding.

Mix, K. S., Prather, R. W., Smith, L. B., & Stockton, J. D. (2014). (See References). Demonstrates that young children possess early, improvable place-value knowledge well before formal schooling.

## Transparency

*Action Editor:* Robert L. Goldstone

*Editor:* Robert L. Goldstone

## Notes

1. We acknowledge that “understanding” is not a unitary construct. Readers seeking further discussion may consult foundational work on conceptual versus procedural knowledge (e.g., Hiebert & Lefevre, 2013; Rittle-Johnson et al., 2015; Rittle-Johnson & Siegler, 2022).

## References

[bibr1-09637214261417968] Bower C. A. Mix K. S. Hancock G. R. Yuan L. Smith L. B. (2024). Smart errors in learning multidigit number meanings. Journal of Cognition and Development, 25(4), 487–510.

[bibr2-09637214261417968] Bower C. A. Mix K. S. Yuan L. Smith L. B. (2022). A network analysis of children’s emerging place-value concepts. Psychological Science, 33(7), 1112–1127. 10.1177/09567976211070242 35699572

[bibr3-09637214261417968] Byrge L. Smith L. B. Mix K. S. (2014). Beginnings of place value: How preschoolers write three-digit numbers. Child Development, 85(2), 437–443. 10.1111/cdev.12162 PMC4445456 24003873

[bibr4-09637214261417968] Chan B. M. Ho C. S. (2010). The cognitive profile of Chinese children with mathematics difficulties. Journal of Experimental Child Psychology, 107(3), 260–279. 10.1016/j.jecp.2010.04.016 20580379

[bibr5-09637214261417968] Chan W. W. L. Au T. K. Tang J. (2014). Strategic counting: A novel assessment of place-value understanding. Learning and Instruction, 29, 78–94.

[bibr6-09637214261417968] Cheung P. Ansari D. (2021). Cracking the code of place value: The relationship between place and value takes years to master. Developmental Psychology, 57(2), 227–240. 10.1037/dev0001145 33370138

[bibr7-09637214261417968] Dehaene S. Sablé-Meyer M. Ciccione L. (2025). Origins of numbers: A shared language-of-thought for arithmetic and geometry? Trends in Cognitive Sciences, 29(6), 526–540. 10.1016/j.tics.2025.03.001 PMC7618345 40234140

[bibr8-09637214261417968] Ferrigno S. Cheyette S. Piantadosi S. T. Cantlon J. F. (2020). Recursive sequence generation in monkeys, children, and native Amazonians. Science Advances, 6, Article eaaz1002. 10.1126/sciadv.aaz1002 PMC7319756 32637593

[bibr9-09637214261417968] Fuson K. C. (1990). A forum for researchers: Issues in place-value and multidigit addition and subtraction learning and teaching for research on mathematics teaching. Journal for Research in Mathematics Education, 21(4), 273–280.

[bibr10-09637214261417968] Fuson K. C. Briars D. J. (1990). Using a base-ten blocks learning/teaching approach for first-and second-grade place-value and multidigit addition and subtraction. Journal for Research in Mathematics Education, 21(3), 180–206.

[bibr11-09637214261417968] Fuson K. C. Wearne D. Hiebert J. C. Murray H. G. Human P. G. Olivier A. I. Carpenter T. P. Fennema E. (1997). Children’s conceptual structures for multidigit numbers and methods of multidigit addition and subtraction. Journal for Research in Mathematics Education, 28(2), 130–162.

[bibr12-09637214261417968] Hanich L. B. Jordan N. C. Kaplan D. Dick J. (2001). Performance across different areas of mathematical cognition in children with learning difficulties. Journal of Educational Psychology, 93(3), 615–626.

[bibr13-09637214261417968] Hauser M. D. Chomsky N. Fitch W. T. (2002). The faculty of language: What is it, who has it, and how did it evolve? Science, 298(5598), 1569–1579. 10.1126/science.298.5598.1569 12446899

[bibr14-09637214261417968] Herzog M. Ehlert A. Fritz A. (2019). Development of a sustainable place value understanding. In Fritz A. Haase V. G. Räsänen P. (Eds.), International handbook of mathematical learning difficulties: From the laboratory to the classroom (pp. 561–579). Springer.

[bibr15-09637214261417968] Herzog M. Fritz A. (2022). Place value understanding explains individual differences in writing numbers in second and third graders but goes beyond. Frontiers in Education, 6, Article 642153. 10.3389/feduc.2021.642153

[bibr16-09637214261417968] Hiebert J. Lefevre P. (2013). Conceptual and procedural knowledge in mathematics: An introductory analysis. In Hiebert J. (Ed.), Conceptual and procedural knowledge (pp. 1–27). Routledge.

[bibr17-09637214261417968] Ho C. S.-H. Cheng F. S.-F. (1997). Training in place-value concepts improves children’s addition skills. Contemporary Educational Psychology, 22(4), 495–506. 10.1006/ceps.1997.0947 9356185

[bibr18-09637214261417968] Jordan N. C. Hanich L. B. (2000). Mathematical thinking in second-grade children with different forms of LD. Journal of Learning Disabilities, 33(6), 567–578. 10.1177/002221940003300605 15495398

[bibr19-09637214261417968] Lafay A. Osana H. P. Levin J. R. (2023). Does conceptual transparency in manipulatives afford place-value understanding in children at risk for mathematics learning disabilities? Learning Disability Quarterly, 46(2), 92–105. 10.1177/07319487221124088 PMC10164236 37168325

[bibr20-09637214261417968] Lambert K. Moeller K. (2019). Place-value computation in children with mathematics difficulties. Journal of Experimental Child Psychology, 178, 214–225. 10.1016/j.jecp.2018.09.008 30390494

[bibr21-09637214261417968] Liao D. A. Brecht K. F. Johnston M. Nieder A. (2022). Recursive sequence generation in crows. Science Advances, 8(44), Article eabq3356. 10.1126/sciadv.abq3356 PMC9629703 36322648

[bibr22-09637214261417968] McCloskey M. (1992). Cognitive mechanisms in numerical processing: Evidence from acquired dyscalculia. Cognition, 44(1–2), 107–157. 10.1016/0010-0277(92)90052-j 1511584

[bibr23-09637214261417968] McGuire P. Kinzie M. B. (2013). Analysis of place value instruction and development in pre-kindergarten mathematics. Early Childhood Education Journal, 41, 355–364.

[bibr24-09637214261417968] Miura I. T. Okamoto Y. (1989). Comparisons of US and Japanese first graders’ cognitive representation of number and understanding of place value. Journal of Educational Psychology, 81(1), 109–114.

[bibr25-09637214261417968] Miura I. T. Okamoto Y. Kim C. C. Steere M. Fayol M. (1993). First graders’ cognitive representation of number and understanding of place value: Cross-national comparisons: France, Japan, Korea, Sweden, and the United States. Journal of Educational Psychology, 85(1), 24–30.

[bibr26-09637214261417968] Mix K. S. Bower C. A. Hancock G. R. Crespo S. Smith L. B. (2024). Place value learning in kindergarten. Journal of Educational Psychology, 116(7), 1129–1152. 10.1037/edu0000866

[bibr27-09637214261417968] Mix K. S. Bower C. A. Hancock G. R. Yuan L. Smith L. B. (2022). The development of place value concepts: Approximation before principles. Child Development, 93(3), 778–793. 10.1111/cdev.13724 PMC9177579 35023576

[bibr28-09637214261417968] Mix K. S. Bower C. A. Yuan L. Hancock G. R. Smith L. B. (2023). Predictive relations between early place value understanding and multidigit calculation: Approximate versus syntactic measures. Educational Psychology, 43(7), 795–813.

[bibr29-09637214261417968] Mix K. S. Prather R. W. Smith L. B. Stockton J. D. (2014). Young children’s interpretation of multidigit number names: From emerging competence to mastery. Child Development, 85(3), 1306–1319. 10.1111/cdev.12197 PMC4460578 24354885

[bibr30-09637214261417968] Moeller K. Pixner S. Zuber J. Kaufmann L. Nuerk H.-C. (2011). Early place-value understanding as a precursor for later arithmetic performance—A longitudinal study on numerical development. Research in Developmental Disabilities, 32(5), 1837–1851. 10.1016/j.ridd.2011.03.012 21498043

[bibr31-09637214261417968] O’Connor P. A. Morsanyi K. McCormack T. (2018). Young children’s non-numerical ordering ability at the start of formal education longitudinally predicts their symbolic number skills and academic achievement in maths. Developmental Science, 21(5), Article e12645. 10.1111/desc.12645 29372580

[bibr32-09637214261417968] Pinker S. Jackendoff R. (2005). The faculty of language: What’s special about it? Cognition, 95(2), 201–236. 10.1016/j.cognition.2004.08.004 15694646

[bibr33-09637214261417968] Rittle-Johnson B. Schneider M. Star J. R. (2015). Not a one-way street: Bidirectional relations between procedural and conceptual knowledge of mathematics. Educational Psychology Review, 27(4), 587–597.

[bibr34-09637214261417968] Rittle-Johnson B. Siegler R. S. (2022). The relation between conceptual and procedural knowledge in learning mathematics: A review. In Donlan C. (Ed.), The development of mathematical skills (pp. 75–110). Psychology Press.

[bibr35-09637214261417968] Ross S. H. (1986). The development of children’s place-value numeration concepts in grades two through five. ERIC. https://eric.ed.gov/?id=ED273482

[bibr36-09637214261417968] Ross S. H. (1989). Parts, wholes, and place value: A developmental view. The Arithmetic Teacher, 36(6), 47–51. 10.5951/at.36.6.0047

[bibr37-09637214261417968] Sella F. Lucangeli D. Kadosh R. C. Zorzi M. (2020). Making sense of number words and Arabic digits: Does order count more? Child Development, 91(5), 1456–1470. 10.1111/cdev.13335 31724163

[bibr38-09637214261417968] Sella F. Lucangeli D. Zorzi M. (2019). Spatial order relates to the exact numerical magnitude of digits in young children. Journal of Experimental Child Psychology, 178, 385–404. 10.1016/j.jecp.2018.09.001 30314720

[bibr39-09637214261417968] Sinclair A. Garin A. Tièche-Christinat C. (1992). Constructing and understanding of place value in numerical notation. European Journal of Psychology of Education, 7, 191–207.

[bibr40-09637214261417968] Yuan L. Prather R. W. Mix K. S. Smith L. B. (2019). Preschoolers and multi-digit numbers: A path to mathematics through the symbols themselves. Cognition, 189, 89–104. 10.1016/j.cognition.2019.03.013 PMC7877708 30933877

[bibr41-09637214261417968] Zhang J. Norman D. A. (1995). A representational analysis of numeration systems. Cognition, 57(3), 271–295. 10.1016/0010-0277(95)00674-3 8556844

